# A single-domain protein catenane of dihydrofolate reductase

**DOI:** 10.1093/nsr/nwad304

**Published:** 2023-11-29

**Authors:** Jing Fang, Tianzuo Li, Jiyeon Lee, Dahye Im, Lianjie Xu, Yajie Liu, Jongcheol Seo, Wen-Bin Zhang

**Affiliations:** Beijing National Laboratory for Molecular Sciences, Key Laboratory of Polymer Chemistry & Physics of Ministry of Education, Center for Soft Matter Science and Engineering, College of Chemistry and Molecular Engineering, Peking University, Beijing 100871, China; Beijing National Laboratory for Molecular Sciences, Key Laboratory of Polymer Chemistry & Physics of Ministry of Education, Center for Soft Matter Science and Engineering, College of Chemistry and Molecular Engineering, Peking University, Beijing 100871, China; Department of Chemistry, Pohang University of Science and Technology (POSTECH), Pohang 37673, Republic of Korea; Department of Chemistry, Pohang University of Science and Technology (POSTECH), Pohang 37673, Republic of Korea; Beijing National Laboratory for Molecular Sciences, Key Laboratory of Polymer Chemistry & Physics of Ministry of Education, Center for Soft Matter Science and Engineering, College of Chemistry and Molecular Engineering, Peking University, Beijing 100871, China; Beijing National Laboratory for Molecular Sciences, Key Laboratory of Polymer Chemistry & Physics of Ministry of Education, Center for Soft Matter Science and Engineering, College of Chemistry and Molecular Engineering, Peking University, Beijing 100871, China; Department of Chemistry, Pohang University of Science and Technology (POSTECH), Pohang 37673, Republic of Korea; Beijing National Laboratory for Molecular Sciences, Key Laboratory of Polymer Chemistry & Physics of Ministry of Education, Center for Soft Matter Science and Engineering, College of Chemistry and Molecular Engineering, Peking University, Beijing 100871, China; Beijing Academy of Artificial Intelligence, Beijing 100084, China

**Keywords:** cellular synthesis, catenane, protein domain, chemical topology, DHFR

## Abstract

A single-domain protein catenane refers to two mechanically interlocked polypeptide rings that fold synergistically into a compact and integrated structure, which is extremely rare in nature. Here, we report a single-domain protein catenane of dihydrofolate reductase (*cat*-DHFR). This design was achieved by rewiring the connectivity between secondary motifs to introduce artificial entanglement and synthesis was readily accomplished through a series of programmed and streamlined post-translational processing events in cells without any additional *in vitro* reactions. The target molecule contained few exogenous motifs and was thoroughly characterized using a combination of ultra-performance liquid chromatography–mass spectrometry, sodium dodecyl sulfate–polyacrylamide gel electrophoresis, protease cleavage experiments and ion mobility spectrometry–mass spectrometry. Compared with the linear control, *cat*-DHFR retained its catalytic capability and exhibited enhanced stability against thermal or chemical denaturation due to conformational restriction. These results suggest that linear proteins may be converted into their concatenated single-domain counterparts with almost identical chemical compositions, well-preserved functions and elevated stabilities, representing an entirely new horizon in protein science.

## Teaser

A single-domain protein catenane of DHFR was designed and directly synthesized in cellular, which raises the *T*_m_ by 6°C relative to the linear control and also has improved aggregation resistance as well as enhanced thermal resilience.

## INTRODUCTION

Nature uses protein domains as modules to tailor existing functions and cultivate new ones because they can exist independently, fold autonomously and evolve dynamically [1]. Natural protein domains are based on linear polypeptide chains, whose genes can be conveniently ‘edited’ and ‘slotted’ into the genome during evolution. Therefore, their backbones exhibit limited topological diversity. The discovery of natural topological proteins, although scarce, suggests that proteins with unconventional chemical topologies could potentially lead to enhanced protein stability compared with their linear counterparts [2–4]. Recently, topology has been recognized as a unique dimension in protein engineering [5–8]. Thanks to the ‘assembly–reaction’ synergy, the design and synthesis of artificial topological proteins have advanced tremendously [4,9], offering access to various topological proteins, including star proteins [10], cyclic proteins [11–19], lasso proteins [20,21], protein catenanes [22–31] and protein pretzelanes [32]. Among them, the protein catenane is composed of two or more mechanically interlocked polypeptide chains with a Hopf link (i.e. [2]catenane) being the simplest form of nontrivial link. This approach often involves the use of genetically encoded entangled reaction motifs. As such, artificial topological proteins synthesized to date usually contain multiple domains, complicating the study of topological effects on structure–property relationships. Ideally, a single-domain topological protein should exhibit the most profound impact of chemical topology and provide an unusual scaffold for further engineering and evolution. However, the preparation of such single-domain topological proteins, even for the Hopf link, remains challenging.

A single protein domain typically has a closely packed hydrophobic core consisting of rigid secondary motifs connected by flexible loops. The highly conserved hydrophobic core allows extensive structural engineering, such as circular permutation [33–36], domain swapping [37–39], loop rethreading [40] and split reconstitution [41,42]. Mutual complementarity among segments preserves structural integrity, despite distinct connectivity. Both green fluorescent protein [43] and dihydrofolate reductase (DHFR) [40] have been shown to tolerate rethreading, producing a linear variant with a swapped sequence that still folds into an overall similar structure with more or less preserved function. Rewiring has also been performed on the SpyTag–SpyCatcher complex, leading to the development of an active template for protein heterocatenane synthesis [26]. Recently, we reported the use of mutually orthogonal split–intein ligation pairs to facilitate the streamlined synthesis of protein heterocatenanes with minimal residue from ligation [22,23]. We envision that the need for an exogenous entangling motif as a template for catenane synthesis may be eliminated by exploiting the spatially entangled relationships among secondary structures and introducing artificial entanglement into the original domain by rewiring the loop region. Using the original fold itself as the entangling template, single-domain protein catenanes may be designed and synthesized, even with a composition that is almost identical to that of the linear wild-type.

In this study, we demonstrate the feasibility of this approach by converting a model enzyme, DHFR, from *Escherichia coli*, into its single-domain catenane counterpart (*cat*-DHFR). DHFR catalyses the reduction of 7,8-dihydrofolate (DHF) to 5,6,7,8-tetrahydrofolate (THF) in the presence of a cofactor, nicotinamide adenine dinucleotide phosphate (NADPH). Despite numerous studies on its engineering and evolution, none goes beyond the linear backbone, as defined by the template polymerization mechanism of the cellular ribosomal machinery. We showed that it is possible to change the chemical topology of a protein domain without collapsing the folds and to bring about profound changes in its properties.

## RESULTS AND DISCUSSION

### Molecular design of *cat*-DHFR

**Figure 1.**
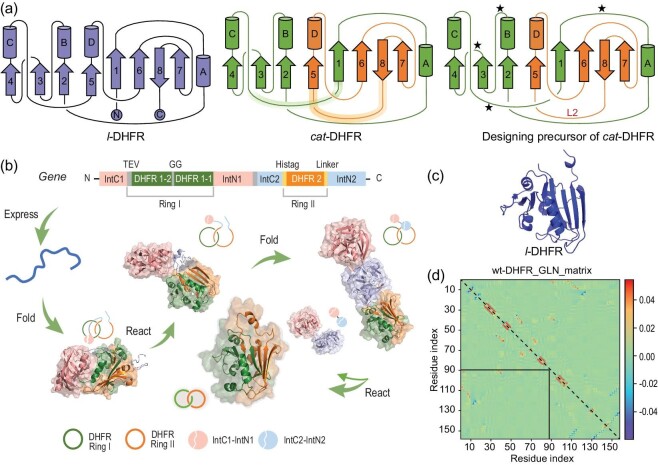
Design and biosynthesis of catenated dihydrofolate reductase (DHFR). (a) Protein topological diagram of *l*-DHFR (left) and *cat*-DHFR (middle), and the retrosynthetic analysis of *cat*-DHFR (right). Numbers 1–8 and letters A–D represent the β-sheet and α-helix, respectively, from N- to C-termini in consecutive order. The split site (Residues 88 and 89) is located at the loop region between α-helix-C and β-sheet-5. The highlighted lines are the linkers generated when forming the *cat*-DHFR. The star denotes the possible position for Ring I closure (at the same and opposite sides) and split–intein insertion. L2 is the linker newly introduced to Ring II of *cat*-DHFR. (b) Scheme of the *cat*-DHFR biosynthesis process using programmed post-translation processing events. DHFR1 is circularly permutated and the corresponding sequences are denoted by DHFR1-1 and DHFR1-2. The TEV recognition site and a GG linker were inserted into Ring I. The His-tag and a variable linker (together, they are L2) were inserted into Ring II. (c) Structure prediction (https://robetta.bakerlab.org/) of *l*-DHFR. (d) The Gauss linking number (GLN) matrix of wt-DHFR. It comprises GLN values between all neighboring residue pairs within the same chain. The sum of all the cells within the boxed submatrix corresponds to the GLN value between the two subchains, which provides a quantitative metric of the extent of their entanglement.

Theoretically, there are numerous ways to create *cat*-DHFR via rewiring wild-type DHFR (wt-DHFR). Taking the virtual loop between the N- and C-termini into consideration and assuming that *M* out of a total of *N* loops are split for rewiring to eventually produce two rings, there is a total of *N*!/[(*N* – *M*)! × *M*!] different splitting methods and a total of $\frac{{N!}}{{( {N{\mathrm{\ }} - {\mathrm{\ }}M} )!{\mathrm{\ }} \times {\mathrm{\ }}M!}} \times \mathop {\sum \nolimits_{L{\mathrm{\ }} = {\mathrm{\ }}1} ^{M{\mathrm{\ }} - {\mathrm{\ }}1}} \frac{{M!}}{{2L( {M{\mathrm{\ }} - {\mathrm{\ }}L} )}}$ different connectivity routes where *L* represents any integer from 1 to *M* – 1. The number rapidly increases with increasing *N* and *M*, among which only those with the correct spatial relationship yield the desired Hopf link. There are even more designs if we consider the variations in the length and sequence of new loops. A successful design should not only ensure an entangled conformation, but also embrace high reconstitution efficiency. For the former, the extent of entanglement was assessed using the Gaussian linking number (GLN) matrix [44] of wt-DHFR (PDB:4KJJ) to identify the splitting position (Fig. 1d). The DHFR split that gives the largest GLN is believed to possess a potentially entangling core, and new loops were then designed with distinct lengths to ensure proper crossing. The GLN matrices of split DHFR and *cat*-DHFR are shown in Supplementary Fig. S1, where the backbone of wt-DHFR was virtually cleaved to generate split segments, with the cleavage site traversing all unstructured loops. The extent of the intertwining of the two subchains was quantitatively evaluated using the GLN. For the latter, we focused on designs with a minimal number of split loops and a higher preference was given to those with proven success in reconstitution reported in the literature. It is expected that the activities of enzymes will not be affected. Hence, if possible, the active site and adjacent regions should be left intact. Combining these criteria, we identified that cleavage at the loop region between α-helix-C and β-sheet-5 (specifically between Residues 88 and 89) would generate two highly intertwined segments (GLN + 0.726, Supplementary Fig. S1a) that could be converted into a catenane topology upon their cyclization. Thus, a design comprising Residues 1–88 as one ring and Residues 89–159 as the other was adopted (Fig. 1a). These two fragments are efficiently reconstituted [42,45]. The entangled core of the design was similar to that of the wild-type and the linker naturally extended to form a ring, giving a *cat*-DHFR with a GLN of +1.120 (Supplementary Fig. S1b). Therefore, we developed a design approach for converting a linear protein into its concatenated form.

### Cellular synthesis of *cat*-DHFR

Streamlined biosynthesis using two pairs of mutually orthogonal split–intein pairs [22,23] provides a straightforward method to synthesize *cat*-DHFR using split DHFR as an entangled template. To ensure the reconstitution and cyclization of the two polypeptide chains while maintaining the correct spatial relationship, the length of the new loop and the sites of ring closure should be carefully chosen, as shown in the retrosynthetic analysis in Fig. 1a. Considering that cyclization efficiency depends on the distance between the new termini and that the two bulky split–intein pairs may interfere with each other, Ring I was circularly permutated to generate new termini between Residues 23 and 24, and the rewired loops were designed to be sufficiently long (16 residues) to bridge the split structural motifs. The gene cassette was designed to contain two DHFR segments as the entangling template, two mutually orthogonal split–intein pairs for ring closure and a tobacco etch virus (TEV) protease recognition sequence in the first ring for subsequent proof of topology by proteolytic digestion (Fig. 1b). A linear DHFR with identical amino-acid composition (*l*-DHFR) was then designed as a linear control bearing extra amino acids not present in the wild-type at the N- and C-termini (Fig. 1a and c; see Supporting Information for sequence information of the two constructs).

**Figure 2.**
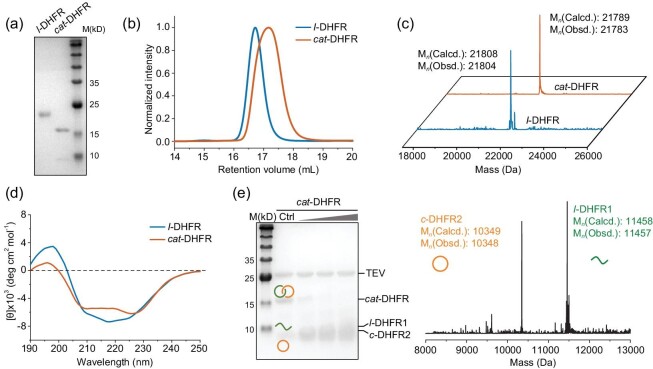
Physiochemical characterizations. (a) SDS–PAGE analysis, (b) SEC traces, (c) LC–MS spectra, (d) Far-UV CD of purified *l*- and *cat*-DHFR. (e) TEV-protease digestion of *cat-*DHFR and mass spectra of the digestion products.

The genes of *cat*- and *l*-DHFR were synthesized and subcloned into pMCSG19 and pET21a vectors, respectively, for expression. Plasmids harboring these genes were used to transform *E. coli* BL21(DE3) competent cells. Protein expression was performed at 16°C for 20 h to ensure proper folding and complete reaction. After cell lysis, the proteins were purified under native conditions using Ni-NTA affinity chromatography. The eluted crude products mainly contained oligomers, catenanes and single-ring ‘unlink’ components. Elution using an elution buffer containing 100 mM of imidazole could significantly enrich the catenane compositions in the mixture. The samples were further purified using size exclusion chromatography (SEC) to remove oligomers and single rings. Sodium dodecyl sulfate–polyacrylamide gel electrophoresis (SDS–PAGE) analysis of the purified products showed a single band at a molecular weight of ∼22 kDa for *l*-DHFR and a major band at <20 kDa for *cat*-DHFR (Fig. 2a). The faint bands at lower molecular weights, attributed to the individual component rings, were reduced to a minimum. The apparent higher mobility of *cat*-DHFR in SDS–PAGE is reasonable because the concatenated form tends to be much more compact than the fully unfolded linear chains. The SEC overlay showed that *cat*-DHFR eluted as a much broader peak at a slightly larger retention volume than *l*-DHFR (Fig. 2b). This is consistent with the SDS–PAGE results showing that *cat*-DHFR has a more compact structure and further suggests that *cat*-DHFR may have a dynamic structure owing to the coupled movement of two mechanically interlocked rings. The identities of *cat*- and *l*-DHFR were confirmed by their experimental molecular weights (*M*_n_,*_cat_*_-DHFR_ = 21 783 Da, *M*_n_,*_l_*_-DHFR_ = 21 804 Da) in ultra-performance liquid chromatography–mass spectrometry (LC–MS) spectra, matching those of the expected calculated values (*M*_n_,*_cat_*_-DHFR_ = 21 789 Da, *M*_n_,*_l_*_-DHFR_ = 21 808 Da) (Fig. 2c). The expression yield of *cat*-DHFR was slightly lower than that of *l*-DHFR because of multistep post-translational modification reactions, which is not surprising because the sequence may not be well fitted for the catenane form. We anticipate that directed evolution may help improve the expression yield of *cat-*DHFR to match that of *l*-DHFR when folding and reaction occur synergistically.

The far-UV CD spectra showed that *cat*-DHFR remained folded yet had substantially reduced intensities of both positive peaks in ellipticity at ∼200 nm and a negative peak in ellipticity at between 210 and 230 nm compared with the linear control. These results suggested that the folded structure of *cat*-DHFR may be more dynamic than the linear version, exhibiting, on average, a reduced proportion of α-helix and β-sheet compositions (Fig. 2d). According to previous studies [45–47], a similar change was observed in circularly permutated linear DHFR. Circular permutations may cause minor local disturbances in local folded structures, but the overall structure remains intact [46]. The near-UV CD spectrum of *cat*-DHFR exhibited positive ellipticity at ∼290 nm, similar to that of the linear control (Supplementary Fig. S2). Although we were unable to obtain the crystal structure of *cat*-DHFR (*vide infra*), the existence of a folded structure in *cat*-DHFR, though slightly deviated from the linear one, is without question, which is also consistent with the role of folding in catenane formation. This is a prerequisite as a template for catenane formation, but it is also reinforced by catenane topology.

Notably, the current approach requires considerable optimization efforts to achieve assembly–reaction synergy for high-yield expression and efficient catenane formation. We also attempted to construct split–intein ligation pairs on the same side of the globular-folded DHFR for ring closure (Supplementary Fig. S3a). However, much fewer catenane products were formed (∼33% in the catenane form), with the majority being single-ring byproducts. Considering that the reconstituted intein domain is very bulky, with >130 residues, it is not surprising to see that they present considerable steric hindrance when placed on the same side of the globular DHFR, affecting the correct reconstitution of DHFR fragments into an intertwined conformation for concatenation. In this case, circular permutation is necessary to place the cyclization motifs on the opposite side of the DHFR, preferably in the loop regions (Fig. 1a; see Supporting Information for sequences). Among these possibilities, the site between Residues 23 and 24 was the most efficient (Supplementary Fig. S3b–d). The length and sequence of newly added loops may also play important roles. In the present study, we selected innocent flexible linkers with embedded elements essential for the purification or proof of topology. To probe the linker effect, we tested L2 of Ring II with different sequences and lengths (Supplementary Fig. S4). As L2 becomes shorter, the split intein cannot efficiently reconstitute and react; therefore, most of the precursors form monocyclic byproducts, leading to a decrease in catenane yield. However, although the yield decreased to a certain extent and the proportion of monocyclic byproducts and oligomers increased slightly, catenated products were still obtained (Supplementary Fig. S4). Notably, the yield with the shortest linker was only approximately one-fifth that with the longest (∼12 mg/L of culture). Hence, this method is generally robust even before the linker becomes extremely short.

### Proof of topology for *cat*-DHFR

Unambiguous proof of topology is obtained by using crystallography. We attempted to crystallize *cat*-DHFR but failed to obtain crystals of sufficient quality. This may be due to unsatisfactory purity, the existence of disordered linkers or reduced binding affinity with the substrates. Thus, we resorted to indirect characterization methods, such as proteolytic digestion experiments, similarly to previous reports [22,23,25,26,30–32]. A TEV-protease recognition site was preprogrammed into the first ring of *cat*-DHFR. Upon cleavage, *cat*-DHFR was converted into linear and cyclic fragments, as expected, which was confirmed by using SDS–PAGE analysis and LC–MS (Fig. 2e). Further evidence was obtained using advanced mass spectrometry (MS)-related techniques, including ion mobility spectrometry (IMS) and collision-induced dissociation (CID). Different topologies often produce distinguishable differences either in the collision cross-section (CCS) values in IMS or the secondary product ions generated from CID [20,32,48]. Thus, both IMS–MS and CID were used to characterize *l*- and *cat*-DHFR.

**Figure 3.**
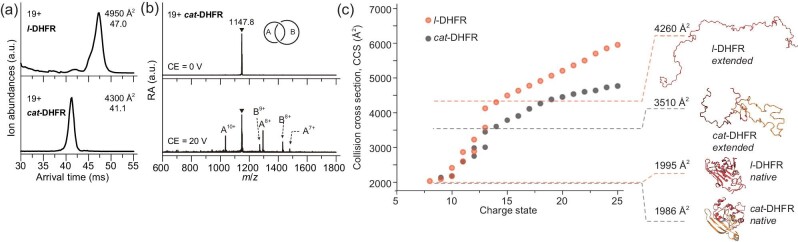
Topology verification of *cat*-DHFR. (a) The arrival time distributions (ATDs) of *l*- and *cat*-DHFR ions at 19+ charge state. The determined CCS values are given together. (b) The product ion mass spectra of 19+ *cat*-DHFR obtained by collision-induced dissociation (CID). (c) Measured CCS values of *l*- and *cat*-DHFR as a function of charge state. Theoretical CCS values and structures of native and extended proteins obtained from molecular dynamics (MD) simulations are shown together.

The arrival time distributions of the 19+ protein ions ([M + 19H]^19+^, M = *l*- or *cat*-DHFR) were compared (Fig. 3a). The 19+ *l*-DHFR ion has an arrival time in IMS of ∼47 ms, while the 19+ *cat*-DHFR ion has a much shorter arrival time (41.1 ms), indicating that the former has a much more extended or unfolded conformation than the latter in the same charge state, consistently with designated topologies. The CCS values determined for 19+ *l*-DHFR and *cat-*DHFR were ∼4950 and ∼4300 Å^2^, respectively. The entire series of CCS values as a function of charge state is shown in Fig. 3c, which shows that *l*-DHFR has >20% larger CCS values than those of *cat*-DHFR at higher charge states (*z* > 13+). The largely extended *l*-DHFR structure predicted by molecular dynamics simulations was also ∼20% larger than that of the extended *cat*-DHFR, which aligns with experimental observations (the simulated unfolding processes of both proteins are shown in Supplementary Movie S1–2). The increase in CCS values after charge state 13+ is due to chain stretching caused by the large charge–charge repulsions. *cat-*DHFR topology was further confirmed by using tandem mass spectrometry. The CID of 19+ *cat*-DHFR (Fig. 3b) yielded abundant product ions that matched the *m*/*z* values of the two individual fragments. The product ions at *m*/*z* 1036, 1295 and 1479 were assigned to the 10+, 8+ and 7+ ions of Ring II fragments, respectively, with molecular weights of ∼10 350 Da, while those at *m*/*z* 1272 and 1430 were assigned to the 9+ and 8+ ions of Ring I fragments, respectively, with molecular weights of ∼11 439 Da. Cleaving either ring in the catenane unwinds the mechanical interlocking and releases both fragments, which can be detected in their monomeric forms in the product ion mass spectrum. These combined results unambiguously prove catenane topology and establish the single-domain features of *cat*-DHFR.

### Properties of *cat*-DHFR

**Figure 4.**
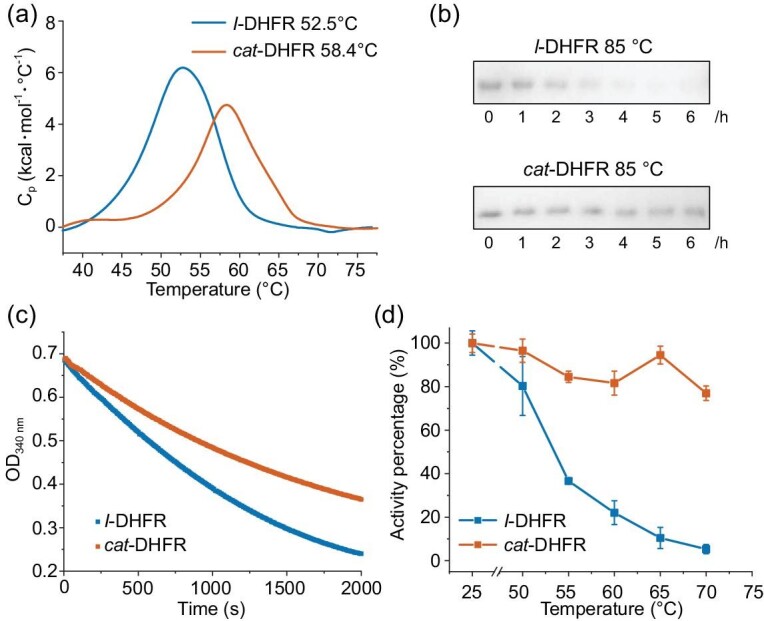
Effects of catenation on DHFR properties. (a) Differential scanning calorimetry characterization and melting temperature (*T*_m_) values for *l*- and *cat*-DHFR. (b) Anti-aggregation properties of *l*- and *cat*-DHFR. The samples (20 μM) were incubated at 85°C for 1–6 h and the supernatant was monitored by using SDS–PAGE. (c) DHFR reduction activity assay of *l*-DHFR (60 nM) and *cat*-DHFR (100 nM). (d) Percentage of catalytic activity preserved for *l*- and *cat*-DHFR after incubation at 50°C, 55°C, 60°C, 65°C and 70°C for 10 min.

We investigated the properties of *cat*-DHFR and its linear counterpart. We first measured the melting temperature (*T*_m_) using differential scanning calorimetry (DSC) to evaluate thermodynamic stability. The *T*_m_ of *cat*-DHFR increased by ∼6°C compared with that of *l*-DHFR (Fig. 4a). However, the ΔH of *l*-DHFR (∼87 kcal/mol) is approximately twice that of *cat*-DHFR (∼49 kcal/mol), indicating that the increase in *T*_m_ is mostly entropic in origin [49,50]. Proteins are prone to aggregation at high temperatures. Notably, *cat*-DHFR exhibited outstanding anti-aggregation properties when incubated at 85°C, whereas *l*-DHFR completely precipitated under similar conditions (Fig. 4b), showcasing the excellent kinetic stability that is highly desirable for enzymes.

Activity is one of the most critical enzyme parameters. The catalytic activity of DHFR was assayed via dihydrofolate reduction with NADPH. The kinetic curves of the DHFR samples were obtained by monitoring the absorbance at 340 nm at 25°C (Fig. 4c). Considering the extensive engineering performed on the structure, it was unsurprising that *cat*-DHFR had lower activity (∼5 U/mg) than that of the linear control (∼14 U/mg). Notably, the previously reported rethreaded DHFR showed no reducing activity [40]. Circular permutations alone can cause a decrease in catalytic activity to varying degrees [45,47]. The reconstituted physical complex of two DHFR fragments (split between α-helix-C and β-sheet-5) retained only 15% of the enzymatic activity of full-length DHFR [45]. Therefore, despite extensive engineering and variations in its sequence and topology, *cat*-DHFR retains a significant proportion of its activity. We envision that reduced activity may be further restored to match, if not surpass, the linear control by directed evolution.

We measured the kinetic parameters *K*_m_ and *k*_cat_ by fitting the Michaelis curve and Hanes–Woolf plots (Supplementary Fig. S7 and Table 1). The *cat*-DHFR has a *K*_m_ that is ∼5-fold that of *l*-DHFR and a *k*_cat_ that is ∼38% that of *l*-DHFR, consistently with reduced activity upon concatenation. The catalytic efficiency (*k*_cat_/*K*_m_) of *cat*-DHFR decreased by an order of magnitude. These results suggest that *cat*-DHFR has a lower affinity toward substrates, a lower turnover rate and decreased catalytic efficiency. To verify the change in affinity, we determined the binding affinities for NADPH, DHF and THF using isothermal titration calorimetry (ITC). As shown in Table 1, the disassociation constants (*K*_D_) were generally much larger for *cat*-DHFR than for *l*-DHFR with the corresponding substrates (∼173-fold for DHF and ∼14-fold for NADPH). Considering that the *K*_m_ of *cat*-DHFR is only 5-fold that of *l*-DHFR at the saturation concentration of NADPH, we assumed that the binding of NADPH significantly enhances the affinity of *cat*-DHFR toward DHF. Notably, the *K*_D_ between *cat*-DHFR and the product THF also increased ∼3.6-fold relative to that of *l*-DHFR, indicating that the product can also easily leave the enzyme. Therefore, the reduced enzymatic activity could be mainly attributed to the compromised affinity of *cat*-DHFR toward the substrate and cofactor.

**Table 1.** Characterization results of dihydrofolate reductase (DHFR) samples.

Protein	*l*-DHFR	*cat*-DHFR
*K*_m_ (μM)	9.4 ± 0.8	44.4 ± 3.7
*k*_cat_ (s^−1^)	6.8 ± 0.3	2.6 ± 0.1
*k*_cat_/*K*_m_ (M^−1^ s^−1^)	(7.1 ± 1.0) × 10^5^	(5.9 ± 0.8) × 10^4^
*K*_D, DHF_ (μM)	2.60 ± 0.63	451 ± 189
*K*_D, NADPH_ (μM)	1.91 ± 0.21	27.4 ± 0.7
*K*_D, THF_ (μM)	102 ± 106	368 ± 313

Despite the compromised activity, catenane topology gained certain functional benefits in addition to increased *T*_m_ and enhanced anti-aggregation. To evaluate the stability of DHFR activity under other conditions, we incubated the DHFR samples at various temperatures for 10 min and then incubated them at 4°C overnight to allow refolding. As shown in Fig. 4d, *cat*-DHFR demonstrates exceptional thermal stability with well-preserved activity (>70%) across the entire range of tested temperatures (≤70°C), whereas *l*-DHFR exhibits a steady decrease in catalytic activity with increasing temperature and is almost completely deactivated at 70°C. The peculiar increase in activity retention at 65°C is probably due to the ‘annealing’ effect at temperatures slightly above the *T*_m_, which promotes refolding upon subsequent cooling. We also tentatively tested the tolerance of the samples to organic solvents by adding DMSO to a final concentration of 5% and performing an activity assay. It seems that *cat*-DHFR retained more activity (87%) than did *l*-DHFR (74%) (Supplementary Fig. S11). Both results suggest that the mechanically interlocking structure facilitates faster refolding of the unfolded DHFR, thus enhancing enzyme resilience. Despite the lower activity of *cat*-DHFR, we anticipate that it will outperform *l*-DHFR at higher temperatures or for longer periods, which is advantageous for the practical application of industrial enzymes.

### Rationale of property enhancement in *cat*-DHFR

Topological proteins exhibit functional benefits related to stabilization. This enhancement is believed to have an entropic origin [49,50]. The conformational restraint imposed by mechanical interlocking and cyclization limits intermolecular interactions and promotes intramolecular interactions, thereby increasing the stability and resilience of the folded structure and making the protein aggregation-resistant [30]. The effect of conformational restriction is better manifested in smaller ring sizes; thus, the single-domain protein catenane is anticipated to exhibit profound topological effects. It should be noted that such properties are also affected by amino-acid composition, detailed sequences and the method of protein splitting [9]. It is hoped that these factors can be synergistically steered toward an optimal functional benefit.

## CONCLUSION

In summary, we designed and synthesized a single-domain protein catenane of DHFR using rewired segments as the entangled template and orthogonal split–inteins for the traceless ligation in cells. The *cat*-DHFR was thoroughly characterized and combined SDS–PAGE, SEC, LC–MS, IMS–MS and proteolytic digestion experiments unambiguously proved its topology. The *cat*-DHFR exhibits enhanced anti-aggregation properties and *T*_m_ by 6°C relative to the linear control. Although the catalytic activity of *cat*-DHFR is reduced owing to its decreased affinity toward the substrate and cofactor, it has better thermal resilience than *l*-DHFR. Even after incubation at 70°C for 10 min, >70% of the catalytic activity was still retained, whereas the linear control lost almost all activity. We anticipate that this method could also be generally applicable to other single-domain proteins, including those with folds similar to DHFR or with completely different folds. The availability of these single-domain protein catenanes facilitates the elucidation of topological effects on structure–property relationships. Our results show that it is possible to construct a single-domain protein catenane from a linear protein precursor with well-preserved functions and additional benefits, opening up new territory for protein molecules. Surpassing the linear paradigm of natural protein molecules, these topological proteins are intrinsically multichain, multidimensional molecules that possess more design space and better evolvability, in addition to the functional benefits of topology. As a new class of protein molecules, they hold great potential for a broad range of applications, including, but not limited to, industrial enzymes, antibodies, cytokines and biomaterials.

## METHODS

### Protein purification and characterizations

Plasmids containing the designed sequences were transformed into *E. coli* strain BL21(DE3) and expressed. After expression, the cells were harvested and lysed by using ultrasonication. The crude products were obtained using Ni-NTA resin and further purified by using SEC. The purified protein samples were characterized by using SDS–PAGE and SEC. The molecular weights of protein samples were confirmed by using LC–MS with a quadrupole rods SQ Detector 2 mass spectrometer (Waters Corp.). Protein samples were diluted with ddH_2_O until a final concentration of 0.02 mg/mL for far-UV CD and 0.2 mg/mL for near-UV CD was achieved. The CD spectroscopy was recorded on a MOS-500 spectrometer (Bio-Logic, France).

### Ion mobility spectrometry–mass spectrometry

The drift-tube ion mobility quadrupole time-of-flight instrument (6560 IM-Q-TOF, Agilent Technologies Inc.) was used for the experiment. The sample solution was injected at a flow rate of 6 μL min^−1^ and sprayed under 3 kV of capillary voltage for positive ion mode. The ions traveled through the drift tube where nitrogen buffer gas was filled under the influence of a weak electric field. The obtained drift time of the specific *m/z* was converted into collision cross-section values using Agilent mass hunter software. For CID analysis, *m/z*-selected ions were accumulated in the collision cell and collided with nitrogen gas.

### Molecular dynamics simulations and collision cross-section calculations

Molecular dynamics (MD) simulations were performed using the OPENMM 7.7 application with the CHARMM force field. Simulations were run under the vacuum condition without any surrounding solvent molecules at a temperature of 1000 K to sample the extended *l*-DHFR and *cat*-DHFR conformers in the gas phase.

### Anti-aggregation experiment

In total, 20 μL of 20-μM samples in phosphate buffered saline (PBS, pH=7.4) were heated at 85°C for 1, 2, 3, 4, 5 and 6 h, respectively, then cooled down to room temperature. The supernatant was collected via centrifugation (12 000 *g*, 10 min), mixed with 5× loading buffer and boiled at 98°C for 10 min.

### DSC and ITC

DSC was carried out on a MicroCal PEAQ-DSC (Malvern Instruments, Inc.). Samples were scanned from 30°C to 95°C with a heating rate of 120°C/h. ITC was carried out on a MicroCal PEAQ-ITC (Malvern Instruments, Inc.). Parameters were set as follows: 19 for total injections, 60 s for initial delay, 750 r/min for stirring speed, 25°C for temperature, 120 s for spacing, 0.4 μL for the first titration and 2 μL for the rest.

### DHFR catalytic activity assay

*l*-DHFR and *cat*-DHFR were diluted using K/Na-phosphate buffer (40.1 mM K_2_HPO_4_, 9.9 mM NaH_2_PO_4_, 5 mM β-mercaptoethanol, pH = 7.5) to 60 and 100 nM, respectively. Then the 100 μL of diluted samples were mixed with 40 μL of NADPH (0.5 mM) and 60 μL of DHF (0.33 mM) in a 96-optical plate. The absorbance at 340 nm of the mixed solution was immediately measured in kinetic mode at 25°C using an EnSpire multimode plate reader (PerkinElmer Inc.). The linear range of all plots was used for the calculation of specific activity.

## Supplementary Material

nwad304_Supplemental_Files
