# Supplementary material for: A single-domain protein catenane of dihydrofolate reductase
Source: Natl Sci Rev. 2023 Nov 29;10(11):nwad304. doi: 10.1093/nsr/nwad304 (PMC10769465; doi:10.1093/nsr/nwad304)
Supplement: nwad304_Supplemental_Files [file nwad304_supplemental_files.zip › SI-NSR 0804.docx]

Supporting Information

A Single-domain Protein Catenane of Dihydrofolate Reductase

Jing Fang^1^, Tianzuo Li^1^, Jiyeon Lee^2^, Dahye Im ^2^, Lianjie Xu^1^, Yajie Liu^1^, Jongcheol Seo^2^, Wen-Bin Zhang^1, 3†^

*^1^ Beijing National Laboratory for Molecular Sciences, Key Laboratory of Polymer Chemistry & Physics of Ministry of Education, Center for Soft Matter Science and Engineering, College of Chemistry and Molecular Engineering, Peking University, Beijing 100871, P. R. China*

*^2^ Department of Chemistry, Pohang University of Science and Technology (POSTECH), Pohang 37673, Republic of Korea*

*^3^ Beijing Academy of Artificial Intelligence, Beijing 100084, P. R. China*

E-mail: wenbin@pku.edu.cn

**Experimental Section**

**GLN matrices calculations.** GLN matrices were calculated according to the adapted formula by Seno et al. [1], with the coordinates of Cα atoms taken as inputs.

**DNA construction.** All oligonucleotide primers were ordered from Genewiz Inc. Sequences encoding DHFR [2] and orthogonal split-intein [3, 4] were amplified by PCR from the corresponding plasmids reported in the previous work. All the genes were treated with standard restriction digestion and ligation protocols for cloning into the expression vector pET21a (for *l*-DHFR) and pMCSG19 (for *cat*-DHFR). All sequences of the genes above were confirmed by direct DNA sequencing.

**Protein expression and purification.** Plasmids containing the designed sequences were transformed into *E.coli* strain BL21(DE3) for the biosynthesis of DHFR with different topologies. The single colony was inoculated in 5 mL of 2xYT medium with 100 μg/mL ampicillin and grew in the shaker (37 ^o^C, 250 rpm) overnight. Then the cultures were inoculated to 300 mL 2xYT medium containing 100 μg/mL ampicillin in a ratio of 1:100 and cultured at 37 ^o^C. The isopropyl β-D-1-thiogalactopyranoside (IPTG) was added to the final concentration of 0.5 mM to induce protein expression when OD600 reached 0.6~1 (for *l*-DHFR, inducing with 1 mM IPTG). The cultures further grew in a shaker at 16 ^o^C for 20 h. After expression, the cells were harvested via centrifugation (6000 rpm, 15 min, 4 ^o^C). The harvested cell pellets were resuspended with 30 mL lysis buffer (50 mM NaH_2_PO_4_, 300 mM NaCl, 10 mM imidazole, pH=8.0) and lysed by ultrasonication. After centrifugation (12000 rpm, 45 min), the supernatant was mixed with Ni-NTA resin (GE Healthcare Inc.) and equilibrated at 4 ^o^C for 1 h. Then the resin was loaded to an empty column, washed by wash buffer (50 mM NaH_2_PO_4_, 300 mM NaCl, 20 mM imidazole, pH=8.0) for several column volumes, and then for *cat*-DHFR eluted by elution buffer 1 (50 mM NaH_2_PO_4_, 300 mM NaCl, 100 mM imidazole, pH=8.0), for *l*-DHFR eluted by elution buffer 2 (50 mM NaH_2_PO_4_, 300 mM NaCl, 250 mM imidazole, pH=8.0). The eluted products were further purified by size exclusion chromatography (SEC) performed on a Superdex 200 Increase 10/300 GL column in an ÄKTA FPLC system (GE Healthcare Inc.), with PBS buffer (137 mM NaCl, 2.7 mM KCl, 10 mM Na_2_HPO_4_, 2 mM KH_2_PO_4_, pH=7.4) as the mobile phase at a flow rate of 0.5 mL/min. Protein concentrations were determined by UV-absorbance with NanoPhotometer NP80 (Implen Inc.).

**Protein characterizations.** The purified protein samples were characterized by sodium dodecyl sulfate-polyacrylamide gel electrophoresis (SDS-PAGE) and SEC. 5 μL of 5 × loading buffer was added to 20 μL of each sample and heated at 98 ^o^C for 10 min before SDS-PAGE analysis. Protein samples’ molecular weights were confirmed by ultra-performance liquid chromatography-mass spectrometry (LC-MS) with quadrupole rods SQ Detector 2 mass spectrometer (Waters Corp.). MassLynx V 4.1 (Waters Corp.) was used to convert the m/z spectrum to the mass spectrum using the MaxEnt1 algorithm (Waters Corp.).

**Protease digestion assay.** Each protein sample was prepared as 20 μM in digestion buffer (1 M Tris-HCl, 2 M NaCl, 20 mM EDTA, 100 mM DTT, pH=8.0 for 20 x buffer) and mixed with TEV protease at the molar ratio of 1:1. Proteolytic digestion experiments were performed in 30 ^o^C and quenched at specific time points by adding 5 x loading buffer and boiling at 98 ^o^C for 10 min. Then the samples were characterized by SDS-PAGE.

**Anti-aggregation experiment.** 20 μL of 20 μM samples in PBS were heated at 85 ^o^C for 1 h, 2 h, 3 h, 4 h, 5 h, and 6 h respectively then cooled down to room temperature. The supernatant was collected via centrifugation (12000 g, 10 min), mixed with 5 x loading buffer, and boiled at 98 ^o^C for 10 min. Then the samples were characterized by SDS-PAGE.

**Circular dichroism spectroscopy.** Protein samples were diluted with ddH_2_O until a final concentration of 0.02 mg/mL for far-UV CD and 0.2 mg/mL for near-UV CD. The CD spectroscopy was recorded on a MOS-500 spectrometer (Bio-Logic., France). Samples were scanned every 1 nm from 190 nm to 250 nm and from 250 nm to 350 nm respectively with a 10 mm path length.

**Differential scanning calorimetry (DSC).** The experiments were carried out on a MicroCal PEAQ-DSC (Malvern Instruments, Inc.). All the samples were diluted to a final concentration of 2 mg/mL in PBS (pH=7.4). Samples were scanned from 30 ^o^C to 95 ^o^C with a heating rate of 120 ^o^C/h. Data were processed using MicroCal Analysis software (Malvern Instruments, Inc.).

**Isothermal titration calorimetry (ITC).** The experiments were carried out on a MicroCal PEAQ-ITC (Malvern Instruments, Inc.). The protein samples were diluted in PBS (pH=7.4) to a final concentration of 50~150 μM for titration. NADPH was diluted in PBS to 0.5~2 mM, DHF was diluted to 0.75~3 mM. THF was diluted to 0.75~2 mM. 40 μL ligand was titrated into a 200 μL protein sample. Parameters were set as followed [5]: 19 for total injections, 60 s for initial delay, 750 rpm for stirring speed, 25 ^o^C for temperature, 120 s for spacing, 0.4 μL for the first titration, and 2 μL for the rest. Data were fitted to a one set of sites model using MicroCal PEAQ-ITC analysis software.

**Ion mobility-mass spectrometry (IM-MS).** The drift-tube ion mobility quadrupole time-of-flight instrument (6560 IM-Q-TOF, Agilent Technologies Inc.) was used for the experiment. The protein samples were prepared 25 μM, dissolved in water/methanol (v/v, 50:50) with 1% of formic acid. 3% of *m*-nitrobenzyl alcohol (m-NBA), as a supercharging reagent, was further added to produce highly charged protein ions. The sample solution was injected with 6 μL min^-1^ flow rate and sprayed under 3 kV capillary voltage for positive ion mode. The ions traveled through the drift tube where nitrogen buffer gas is filled under the influence of weak electric field. Obtained drift time of specific *m/z* was converted into collision-cross section values using Agilent mass hunter software. For collision induced dissociation analysis, *m/z*-selected ions were accumulated in the collision cell and collided with nitrogen gas. By increasing collision energy, fragment ions were actively produced and detected at the time-of-flight analyzer.

**Molecular dynamics simulations and collision cross section calculations.** Molecular dynamics (MD) simulations were performed using OPENMM 7.7 application [6] with the CHARMM force field [7]. Simulations were run under the vacuum condition without any surrounding solvent molecules at 1000 K temperature to sample the extended *l*-DHFR and *cat*-DHFR conformers in the gas phase. Simulation time step was 1.0 fs and protein structures were sampled in every 10 ps. The collision cross section (CCS) values of sampled proteins were calculated by projection approximation method implemented in Sigma program. The average CCS values from the group of the extended conformers were also determined.

**DHFR catalytic activity assay.** The procedure was carried out according to the protocol reported [8, 9]. *l*-DHFR and *cat*-DHFR were diluted with KHP buffer (40.1 mM K_2_HPO_4_, 9.9 mM NaH_2_PO_4_, 5 mM β-mercaptoethanol，pH=7.5) to 60 nM and 100 nM respectively. DMSO was added to the protein samples to a final concentration of 5% for the chemical denaturant experiment. Then the 100 μL diluted samples were mixed with 40 μL NADPH (0.5 mM) and 60 μL DHF (0.33 mM) in a 96-optical plate. The absorbance at 340 nm of the mixed solution was immediately measured in kinetic mode at 25 ^o^C using an EnSpire multimode plate reader (PerkinElmer Inc.). The absorbance at 340 nm of 200 μL KHP buffer containing NADPH (0 nmol, 10 nmol, 20 nmol, 30 nmol, 40 nmol, 60 nmol, and 100 nmol) was plotted and fitted as a standard curve to calculate the amounts of oxidized NADPH during the reaction. The initial linear region of the kinetic curve was fitted, then the slope was divided by that of the standard curve and the enzyme concentration, and converted by the time (min) and mass (mg) to obtain the enzyme activity value.

**Enzymatic kinetic parameters measurements.** For *l*-DHFR, the final concentration was 15 nM. The concentration of a ligand (DHF or NADPH) varied from 2 to 50 μM while the other is 100 μM. For *cat*-DHFR, the final concentration was 50 nM. The concentration of a ligand (DHF or NADPH) varied from 2 to 300 μM while the other is 200 μM. The absorbance at 340 nm of the mixed solution was immediately measured in kinetic mode at 25 ^o^C using an EnSpire multimode plate reader (PerkinElmer Inc.). The NADPH standard curve was also plotted. The slope of the linear range was calculated to make a concentration of substrate/reaction rate – concentration of substrate plot (Hanes-Woolf plot) and the *K*_m_ and *k*_cat_ of DHFR variants were obtained [10, 11].

**Amino acid sequence information**. The amino acid sequences of *l*-DHFR and a series of *cat*-DHFR are listed below. The DHFR, TEV protease recognition sequences, IntC1 and IntN1, IntC2 and Int N2 are colored as green, red, beige, and blue respectively. The final products of *cat*-DHFR after streamlined post-translational processing events are underlined. The shortened L2 linkers are in italics.

***cat*-DHFR**

**001 MKGSSIKIATRKYLGKQNVYDIGVERDHNFALKNGFIASNCFNGGENLYFQGASLPADLA**

**061 WFKRNTLNKPVIMGRHTWESIGRPLPGRKNIILSSQPGTDDRVTWVKSVDEAIAACGDVG**

**121 GMISLIAALAVDRVIGMENAMPWNGTCLSYETEILTVEYGLLPIGKIVEKRIECTVYSVD**

**181 NNGNIYTQPVAQWHDRGEQEVFEYCLEDGSLIRATKDHKFMTVDGQMLPIDEIFERELDL**

**241 MRVDNLPNVDSGSGETVRFQGGGSGGSSGDHNFALKNGFIASNCFNGGHHHHHHELPEIM**

**301 VIGGGRVYEQFLPKAQKLYLTHIDAEVEGDTHFPDYEPDDWESVFSEFHDADAQNSHSYC**

**361 FEILERRGGSGGTSCLSYETEILTVEYGLLPIGKIVEKRIECTVYSVDNNGNIYTQPVAQ**

**421 WHDRGEQEVFEYCLEDGSLIRATKDHKFMTVDGQMLPIDEIFERELDLMRVDNLPNIKIA**

**481 TRKYLGKQNVYDIGVER**

***l*-DHFR(-Met)**

**001 GTCFNGGHHHHHHELGGENLYFQGASMISLIAALAVDRVIGMENAMPWNLPADLAWFKRN**

**061 TLNKPVIMGRHTWESIGRPLPGRKNIILSSQPGTDDRVTWVKSVDEAIAACGDVPEIMVI**

**121 GGGRVYEQFLPKAQKLYLTHIDAEVEGDTHFPDYEPDDWESVFSEFHDADAQNSHSYCFE**

**181 ILERRTSCFNGGGGSGG**

*Trial with ring closure at the same side*

***cat*-DHFR-ss**

**001 MKGSSIKIATRKYLGKQNVYDIGVERDHNFALKNGFIASNCFNGGENLYFQGASMISLIA**

**061 ALAVDRVIGMENAMPWNLPADLAWFKRNTLNKPVIMGRHTWESIGRPLPGRKNIILSSQP**

**121 GTDDRVTWVKSVDEAIAACGGTCLSYETEILTVEYGLLPIGKIVEKRIECTVYSVDNNGN**

**181 IYTQPVAQWHDRGEQEVFEYCLEDGSLIRATKDHKFMTVDGQMLPIDEIFERELDLMRVD**

**241 NLPNVDSGSGETVRFQGGGSGGSSGDHNFALKNGFIASNCFNGGHHHHHHELDVPEIMVI**

**301 GGGRVYEQFLPKAQKLYLTHIDAEVEGDTHFPDYEPDDWESVFSEFHDADAQNSHSYCFE**

**361 ILERRGGSGGTSCLSYETEILTVEYGLLPIGKIVEKRIECTVYSVDNNGNIYTQPVAQWH**

**421 DRGEQEVFEYCLEDGSLIRATKDHKFMTVDGQMLPIDEIFERELDLMRVDNLPNIKIATR**

**481 KYLGKQNVYDIGVER**

*Trial with ring closure at the opposite side with different positions of circular permutation and TEV recognition site*

***cat*-DHFR(24/23) I**

**001 MKGSSIKIATRKYLGKQNVYDIGVERDHNFALKNGFIASNCFNGGENLYFQGASLPADLA**

**061 WFKRNTLNKPVIMGRHTWESIGRPLPGRKNIILSSQPGTDDRVTWVKSVDEAIAACGDVG**

**121 GMISLIAALAVDRVIGMENAMPWNGTCLSYETEILTVEYGLLPIGKIVEKRIECTVYSVD**

**181 NNGNIYTQPVAQWHDRGEQEVFEYCLEDGSLIRATKDHKFMTVDGQMLPIDEIFERELDL**

**241 MRVDNLPNVDSGSGETVRFQGGGSGGSSGDHNFALKNGFIASNCFNGGHHHHHHELPEIM**

**301 VIGGGRVYEQFLPKAQKLYLTHIDAEVEGDTHFPDYEPDDWESVFSEFHDADAQNSHSYC**

**361 FEILERRGGSGGTSCLSYETEILTVEYGLLPIGKIVEKRIECTVYSVDNNGNIYTQPVAQ**

**421 WHDRGEQEVFEYCLEDGSLIRATKDHKFMTVDGQMLPIDEIFERELDLMRVDNLPNIKIA**

**481 TRKYLGKQNVYDIGVER**

***cat*-DHFR(24/23) II**

**001 MKGSSIKIATRKYLGKQNVYDIGVERDHNFALKNGFIASNCFNASLPADLAWFKRNTLNK**

**061 PVIMGRHTWESIGRPLPGRKNIILSSQPGTDDRVTWVKSVDEAIAACGDVGGMISLIAAL**

**121 AVDRVIGMENAMPWNGTCLSYETEILTVEYGLLPIGKIVEKRIECTVYSVDNNGNIYTQP**

**181 VAQWHDRGEQEVFEYCLEDGSLIRATKDHKFMTVDGQMLPIDEIFERELDLMRVDNLPNV**

**241 DSGSGETVRFQGGGSGGSSGDHNFALKNGFIASNCFNGGHHHHHHELPEIMVIGGGRVYE**

**301 QFLPKAQKLYLTHIDAEVEGDTHFPDYEPDDWESVFSEFHDADAQNSHSYCFEILERRGG**

**361 ENLYFQGTSCLSYETEILTVEYGLLPIGKIVEKRIECTVYSVDNNGNIYTQPVAQWHDRG**

**421 EQEVFEYCLEDGSLIRATKDHKFMTVDGQMLPIDEIFERELDLMRVDNLPNIKIATRKYL**

**481 GKQNVYDIGVER**

***cat*-DHFR(54/53) I**

**001 MKGSSIKIATRKYLGKQNVYDIGVERDHNFALKNGFIASNCFNGGENLYFQGASLPGRKN**

**061 IILSSQPGTDDRVTWVKSVDEAIAACGDVGGMISLIAALAVDRVIGMENAMPWNLPADLA**

**121 WFKRNTLNKPVIMGRHTWESIGRPGTCLSYETEILTVEYGLLPIGKIVEKRIECTVYSVD**

**181 NNGNIYTQPVAQWHDRGEQEVFEYCLEDGSLIRATKDHKFMTVDGQMLPIDEIFERELDL**

**241 MRVDNLPNVDSGSGETVRFQGGGSGGSSGDHNFALKNGFIASNCFNGGHHHHHHELPEIM**

**301 VIGGGRVYEQFLPKAQKLYLTHIDAEVEGDTHFPDYEPDDWESVFSEFHDADAQNSHSYC**

**361 FEILERRGGSGGTSCLSYETEILTVEYGLLPIGKIVEKRIECTVYSVDNNGNIYTQPVAQ**

**421 WHDRGEQEVFEYCLEDGSLIRATKDHKFMTVDGQMLPIDEIFERELDLMRVDNLPNIKIA**

**481 TRKYLGKQNVYDIGVER**

***cat*-DHFR(54/53) II**

**001 MKGSSIKIATRKYLGKQNVYDIGVERDHNFALKNGFIASNCFNASLPGRKNIILSSQPGT**

**061 DDRVTWVKSVDEAIAACGDGGMISLIAALAVDRVIGMENAMPWNLPADLAWFKRNTLNK**

**121 PVIMGRHTWESIGRPGTCLSYETEILTVEYGLLPIGKIVEKRIECTVYSVDNNGNIYTQP**

**181 VAQWHDRGEQEVFEYCLEDGSLIRATKDHKFMTVDGQMLPIDEIFERELDLMRVDNLPNV**

**241 DSGSGETVRFQGGGSGGSSGDHNFALKNGFIASNCFNGGHHHHHHELPEIMVIGGGRVYE**

**301 QFLPKAQKLYLTHIDAEVEGDTHFPDYEPDDWESVFSEFHDADAQNSHSYCFEILERRGG**

**361 ENLYFQGTSCLSYETEILTVEYGLLPIGKIVEKRIECTVYSVDNNGNIYTQPVAQWHDRG**

**421 EQEVFEYCLEDGSLIRATKDHKFMTVDGQMLPIDEIFERELDLMRVDNLPNIKIATRKYL**

**481 GKQNVYDIGVER**

***cat*-DHFR(72/71) I**

**001 MKGSSIKIATRKYLGKQNVYDIGVERDHNFALKNGFIASNCFNGGENLYFQGASVTWVKS**

**061 VDEAIAACGDVGGMISLIAALAVDRVIGMENAMPWNLPADLAWFKRNTLNKPVIMGRHTW**

**121 ESIGRPLPGRKNIILSSQPGTDDRGTCLSYETEILTVEYGLLPIGKIVEKRIECTVYSVD**

**181 NNGNIYTQPVAQWHDRGEQEVFEYCLEDGSLIRATKDHKFMTVDGQMLPIDEIFERELDL**

**241 MRVDNLPNVDSGSGETVRFQGGGSGGSSGDHNFALKNGFIASNCFNGGHHHHHHELPEIM**

**301 VIGGGRVYEQFLPKAQKLYLTHIDAEVEGDTHFPDYEPDDWESVFSEFHDADAQNSHSYC**

**361 FEILERRGGSGGTSCLSYETEILTVEYGLLPIGKIVEKRIECTVYSVDNNGNIYTQPVAQ**

**421 WHDRGEQEVFEYCLEDGSLIRATKDHKFMTVDGQMLPIDEIFERELDLMRVDNLPNIKIA**

**481 TRKYLGKQNVYDIGVER**

***cat*-DHFR(72/71) II**

**001 MKGSSIKIATRKYLGKQNVYDIGVERDHNFALKNGFIASNCFNASVTWVKSVDEAIAACG**

**061 DVGGMISLIAALAVDRVIGMENAMPWNLPADLAWFKRNTLNKPVIMGRHTWESIGRPLPG**

**121 RKNIILSSQPGTDDRGTCLSYETEILTVEYGLLPIGKIVEKRIECTVYSVDNNGNIYTQP**

**181 VAQWHDRGEQEVFEYCLEDGSLIRATKDHKFMTVDGQMLPIDEIFERELDLMRVDNLPNV**

**241 DSGSGETVRFQGGGSGGSSGDHNFALKNGFIASNCFNGGHHHHHHELPEIMVIGGGRVYE**

**301 QFLPKAQKLYLTHIDAEVEGDTHFPDYEPDDWESVFSEFHDADAQNSHSYCFEILERRGG**

**361 ENLYFQGTSCLSYETEILTVEYGLLPIGKIVEKRIECTVYSVDNNGNIYTQPVAQWHDRG**

**421 EQEVFEYCLEDGSLIRATKDHKFMTVDGQMLPIDEIFERELDLMRVDNLPNIKIATRKYL**

**481 GKQNVYDIGVER**

*Trial with ring closure at the opposite side but different linker lengths*

***cat*-DHFR-L2-1**

**001 MKGSSIKIATRKYLGKQNVYDIGVERDHNFALKNGFIASNCFNLPADLAWFKRNTLNKPV**

**061 IMGRHTWESIGRPLPGRKNIILSSQPGTDDRVTWVKSVDEAIAACGDVGGMISLIAALAV**

**121 DRVIGMENAMPWNGTCLSYETEILTVEYGLLPIGKIVEKRIECTVYSVDNNGNIYTQPVA**

**181 QWHDRGEQEVFEYCLEDGSLIRATKDHKFMTVDGQMLPIDEIFERELDLMRVDNLPNVDS**

**241 GSGETVRFQGGGSGGSSGDHNFALKNGFIASN*CFNGGHHHHHHEL*PEIMVIGGGRVYEQF**

**301 LPKAQKLYLTHIDAEVEGDTHFPDYEPDDWESVFSEFHDADAQNSHSYCFEILERR*GGSG***

**361 *GTS*CLSYETEILTVEYGLLPIGKIVEKRIECTVYSVDNNGNIYTQPVAQWHDRGEQEVFE**

**421 YCLEDGSLIRATKDHKFMTVDGQMLPIDEIFERELDLMRVDNLPNIKIATRKYLGKQNVY**

**481 DIGVER**

***cat*-DHFR-L2-2**

**001 MKGSSIKIATRKYLGKQNVYDIGVERDHNFALKNGFIASNCFNLPADLAWFKRNTLNKPV**

**061 IMGRHTWESIGRPLPGRKNIILSSQPGTDDRVTWVKSVDEAIAACGDVGGMISLIAALAV**

**121 DRVIGMENAMPWNGTCLSYETEILTVEYGLLPIGKIVEKRIECTVYSVDNNGNIYTQPVA**

**181 QWHDRGEQEVFEYCLEDGSLIRATKDHKFMTVDGQMLPIDEIFERELDLMRVDNLPNVDS**

**241 GSGETVRFQGGGSGGSSGDHNFALKNGFIASN*CFNGGHHHHHHEL*PEIMVIGGGRVYEQF**

**301 LPKAQKLYLTHIDAEVEGDTHFPDYEPDDWESVFSEFHDADAQNSHSYCFEILERR*GSGG***

**361 *TS*CLSYETEILTVEYGLLPIGKIVEKRIECTVYSVDNNGNIYTQPVAQWHDRGEQEVFEY**

**421 CLEDGSLIRATKDHKFMTVDGQMLPIDEIFERELDLMRVDNLPNIKIATRKYLGKQNVYD**

**481 IGVER**

***cat*-DHFR-L2-3**

**001 MKGSSIKIATRKYLGKQNVYDIGVERDHNFALKNGFIASNCFNLPADLAWFKRNTLNKPV**

**061 IMGRHTWESIGRPLPGRKNIILSSQPGTDDRVTWVKSVDEAIAACGDVGGMISLIAALAV**

**121 DRVIGMENAMPWNGTCLSYETEILTVEYGLLPIGKIVEKRIECTVYSVDNNGNIYTQPVA**

**181 QWHDRGEQEVFEYCLEDGSLIRATKDHKFMTVDGQMLPIDEIFERELDLMRVDNLPNVDS**

**241 GSGETVRFQGGGSGGSSGDHNFALKNGFIASN*CFNGGHHHHHHEL*PEIMVIGGGRVYEQF**

**301 LPKAQKLYLTHIDAEVEGDTHFPDYEPDDWESVFSEFHDADAQNSHSYCFEILERR*SGGT***

**361 *S*CLSYETEILTVEYGLLPIGKIVEKRIECTVYSVDNNGNIYTQPVAQWHDRGEQEVFEYC**

**421 LEDGSLIRATKDHKFMTVDGQMLPIDEIFERELDLMRVDNLPNIKIATRKYLGKQNVYDI**

**481 GVER**

***cat*-DHFR-L2-4**

**001 MKGSSIKIATRKYLGKQNVYDIGVERDHNFALKNGFIASNCFNLPADLAWFKRNTLNKPV**

**061 IMGRHTWESIGRPLPGRKNIILSSQPGTDDRVTWVKSVDEAIAACGDVGGMISLIAALAV**

**121 DRVIGMENAMPWNGTCLSYETEILTVEYGLLPIGKIVEKRIECTVYSVDNNGNIYTQPVA**

**181 QWHDRGEQEVFEYCLEDGSLIRATKDHKFMTVDGQMLPIDEIFERELDLMRVDNLPNVDS**

**241 GSGETVRFQGGGSGGSSGDHNFALKNGFIASN*CFNGGHHHHHHEL*PEIMVIGGGRVYEQF**

**301 LPKAQKLYLTHIDAEVEGDTHFPDYEPDDWESVFSEFHDADAQNSHSYCFEILERR*GGTS***

**361 CLSYETEILTVEYGLLPIGKIVEKRIECTVYSVDNNGNIYTQPVAQWHDRGEQEVFEYCL**

**421 EDGSLIRATKDHKFMTVDGQMLPIDEIFERELDLMRVDNLPNIKIATRKYLGKQNVYDIG**

**481 VER**

***cat*-DHFR-L2-5**

**001 MKGSSIKIATRKYLGKQNVYDIGVERDHNFALKNGFIASNCFNLPADLAWFKRNTLNKPV**

**061 IMGRHTWESIGRPLPGRKNIILSSQPGTDDRVTWVKSVDEAIAACGDVGGMISLIAALAV**

**121 DRVIGMENAMPWNGTCLSYETEILTVEYGLLPIGKIVEKRIECTVYSVDNNGNIYTQPVA**

**181 QWHDRGEQEVFEYCLEDGSLIRATKDHKFMTVDGQMLPIDEIFERELDLMRVDNLPNVDS**

**241 GSGETVRFQGGGSGGSSGDHNFALKNGFIASN*CFNGGHHHHHHEL*PEIMVIGGGRVYEQF**

**301 LPKAQKLYLTHIDAEVEGDTHFPDYEPDDWESVFSEFHDADAQNSHSYCFEILERR*GTS*C**

**361 LSYETEILTVEYGLLPIGKIVEKRIECTVYSVDNNGNIYTQPVAQWHDRGEQEVFEYCLE**

**421 DGSLIRATKDHKFMTVDGQMLPIDEIFERELDLMRVDNLPNIKIATRKYLGKQNVYDIGV**

**481 ER**

***cat*-DHFR-L2-6**

**001 MKGSSIKIATRKYLGKQNVYDIGVERDHNFALKNGFIASNCFNLPADLAWFKRNTLNKPV**

**061 IMGRHTWESIGRPLPGRKNIILSSQPGTDDRVTWVKSVDEAIAACGDVGGMISLIAALAV**

**121 DRVIGMENAMPWNGTCLSYETEILTVEYGLLPIGKIVEKRIECTVYSVDNNGNIYTQPVA**

**181 QWHDRGEQEVFEYCLEDGSLIRATKDHKFMTVDGQMLPIDEIFERELDLMRVDNLPNVDS**

**241 GSGETVRFQGGGSGGSSGDHNFALKNGFIASN*CFNGGHHHHHHEL*PEIMVIGGGRVYEQF**

**301 LPKAQKLYLTHIDAEVEGDTHFPDYEPDDWESVFSEFHDADAQNSHSYCFEILERR*TS*CL**

**361 SYETEILTVEYGLLPIGKIVEKRIECTVYSVDNNGNIYTQPVAQWHDRGEQEVFEYCLED**

**421 GSLIRATKDHKFMTVDGQMLPIDEIFERELDLMRVDNLPNIKIATRKYLGKQNVYDIGVE**

**481 R**

***cat*-DHFR-L2-7**

**001 MKGSSIKIATRKYLGKQNVYDIGVERDHNFALKNGFIASNCFNLPADLAWFKRNTLNKPV**

**061 IMGRHTWESIGRPLPGRKNIILSSQPGTDDRVTWVKSVDEAIAACGDVGGMISLIAALAV**

**121 DRVIGMENAMPWNGTCLSYETEILTVEYGLLPIGKIVEKRIECTVYSVDNNGNIYTQPVA**

**181 QWHDRGEQEVFEYCLEDGSLIRATKDHKFMTVDGQMLPIDEIFERELDLMRVDNLPNVDS**

**241 GSGETVRFQGGGSGGSSGDHNFALKNGFIASN*CFNGGHHHHHH*PEIMVIGGGRVYEQFLP**

**301 KAQKLYLTHIDAEVEGDTHFPDYEPDDWESVFSEFHDADAQNSHSYCFEILERR*GGSGGT***

**361 *S*CLSYETEILTVEYGLLPIGKIVEKRIECTVYSVDNNGNIYTQPVAQWHDRGEQEVFEYC**

**421 LEDGSLIRATKDHKFMTVDGQMLPIDEIFERELDLMRVDNLPNIKIATRKYLGKQNVYDI**

**481 GVER**

***cat*-DHFR-L2-8**

**001 MKGSSIKIATRKYLGKQNVYDIGVERDHNFALKNGFIASNCFNLPADLAWFKRNTLNKPV**

**061 IMGRHTWESIGRPLPGRKNIILSSQPGTDDRVTWVKSVDEAIAACGDVGGMISLIAALAV**

**121 DRVIGMENAMPWNGTCLSYETEILTVEYGLLPIGKIVEKRIECTVYSVDNNGNIYTQPVA**

**181 QWHDRGEQEVFEYCLEDGSLIRATKDHKFMTVDGQMLPIDEIFERELDLMRVDNLPNVDS**

**241 GSGETVRFQGGGSGGSSGDHNFALKNGFIASN*CFNHHHHHH*PEIMVIGGGRVYEQFLPKA**

**301 QKLYLTHIDAEVEGDTHFPDYEPDDWESVFSEFHDADAQNSHSYCFEILERR*GGSGGTS*C**

**361 LSYETEILTVEYGLLPIGKIVEKRIECTVYSVDNNGNIYTQPVAQWHDRGEQEVFEYCLE**

**421 DGSLIRATKDHKFMTVDGQMLPIDEIFERELDLMRVDNLPNIKIATRKYLGKQNVYDIGV**

**481 ER**

***cat*-DHFR-L2-9**

**001 MKGSSIKIATRKYLGKQNVYDIGVERDHNFALKNGFIASNCFNLPADLAWFKRNTLNKPV**

**061 IMGRHTWESIGRPLPGRKNIILSSQPGTDDRVTWVKSVDEAIAACGDVGGMISLIAALAV**

**121 DRVIGMENAMPWNGTCLSYETEILTVEYGLLPIGKIVEKRIECTVYSVDNNGNIYTQPVA**

**181 QWHDRGEQEVFEYCLEDGSLIRATKDHKFMTVDGQMLPIDEIFERELDLMRVDNLPNVDS**

**241 GSGETVRFQGGGSGGSSGDHNFALKNGFIASN*CFNGGHHHHHH*PEIMVIGGGRVYEQFLP**

**301 KAQKLYLTHIDAEVEGDTHFPDYEPDDWESVFSEFHDADAQNSHSYCFEILERR*TS*CLSY**

**361 ETEILTVEYGLLPIGKIVEKRIECTVYSVDNNGNIYTQPVAQWHDRGEQEVFEYCLEDGS**

**421 LIRATKDHKFMTVDGQMLPIDEIFERELDLMRVDNLPNIKIATRKYLGKQNVYDIGVER**

***cat*-DHFR-L2-10**

**001 MKGSSIKIATRKYLGKQNVYDIGVERDHNFALKNGFIASNCFNLPADLAWFKRNTLNKPV**

**061 IMGRHTWESIGRPLPGRKNIILSSQPGTDDRVTWVKSVDEAIAACGDVGGMISLIAALAV**

**121 DRVIGMENAMPWNGTCLSYETEILTVEYGLLPIGKIVEKRIECTVYSVDNNGNIYTQPVA**

**181 QWHDRGEQEVFEYCLEDGSLIRATKDHKFMTVDGQMLPIDEIFERELDLMRVDNLPNVDS**

**241 GSGETVRFQGGGSGGSSGDHNFALKNGFIASN*CFNHHHHHH*PEIMVIGGGRVYEQFLPKA**

**301 QKLYLTHIDAEVEGDTHFPDYEPDDWESVFSEFHDADAQNSHSYCFEILERR*TS*CLSYET**

**361 EILTVEYGLLPIGKIVEKRIECTVYSVDNNGNIYTQPVAQWHDRGEQEVFEYCLEDGSLI**

**421 RATKDHKFMTVDGQMLPIDEIFERELDLMRVDNLPNIKIATRKYLGKQNVYDIGVER**


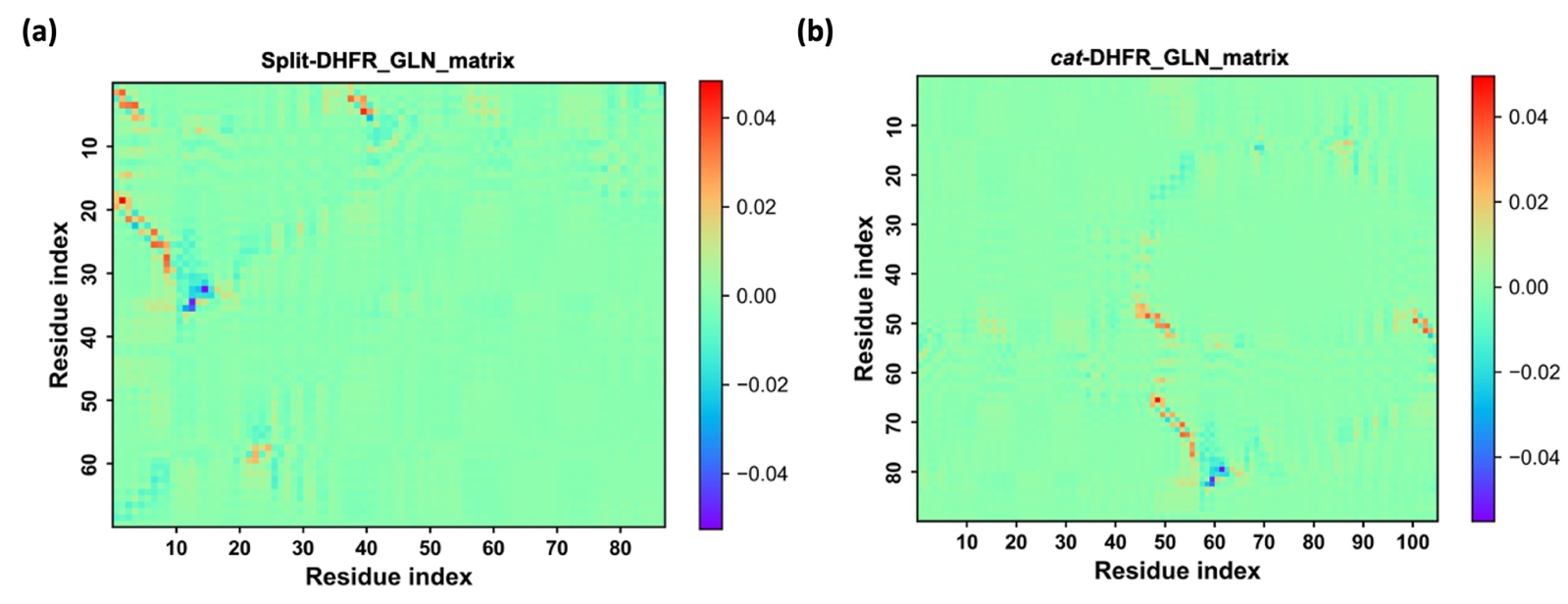


**Figure S1.** The GLN matrix of (a) the split DHFR and (b) the *cat*-DHFR. For the split DHFR, the backbone was cleaved between residues 88 and 89. For the *cat*-DHFR, the sequences of newly introduced loops are shown in the sequence information.





**Figure S2.** Near-UV CD of *l*-DHFR and *cat*-DHFR.


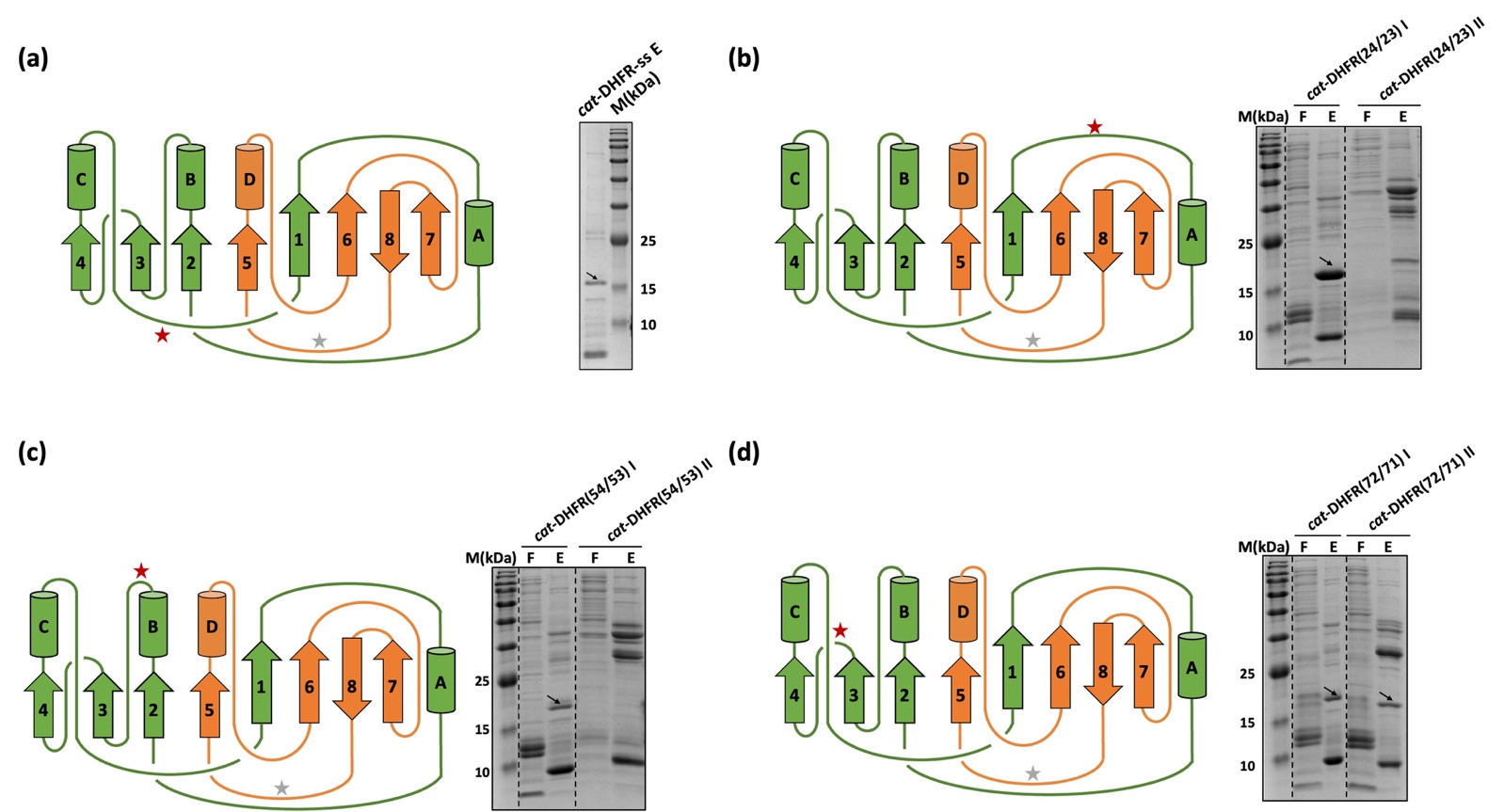


**Figure S3.** Protein topological diagram and SDS-PAGE analysis of *cat*-DHFR with different ring-closing positions. (a) *cat*-DHFR with orthogonal ligation pairs on the same side. The red star denoted the ring closure position of ring I and the grey star denoted that of ring II. The *cat*-DHFR with orthogonal ligation pairs on the opposite side but in different positions (b-d). For *cat*-DHFR(24/23) I, *cat*-DHFR(54/53) I, and *cat*-DHFR(72/71) I, the TEV recognition sequence is inserted into ring I. For *cat*-DHFR(24/23) II, *cat*-DHFR(54/53) II, and *cat*-DHFR(72/71) II, the TEV recognition sequence is inserted into ring II. The black arrows refer to the corresponding concatenated products. *cat*-DHFR(24/23) I is the one with the highest yield, and in the subsequent experiments, it is denoted by *cat*-DHFR.


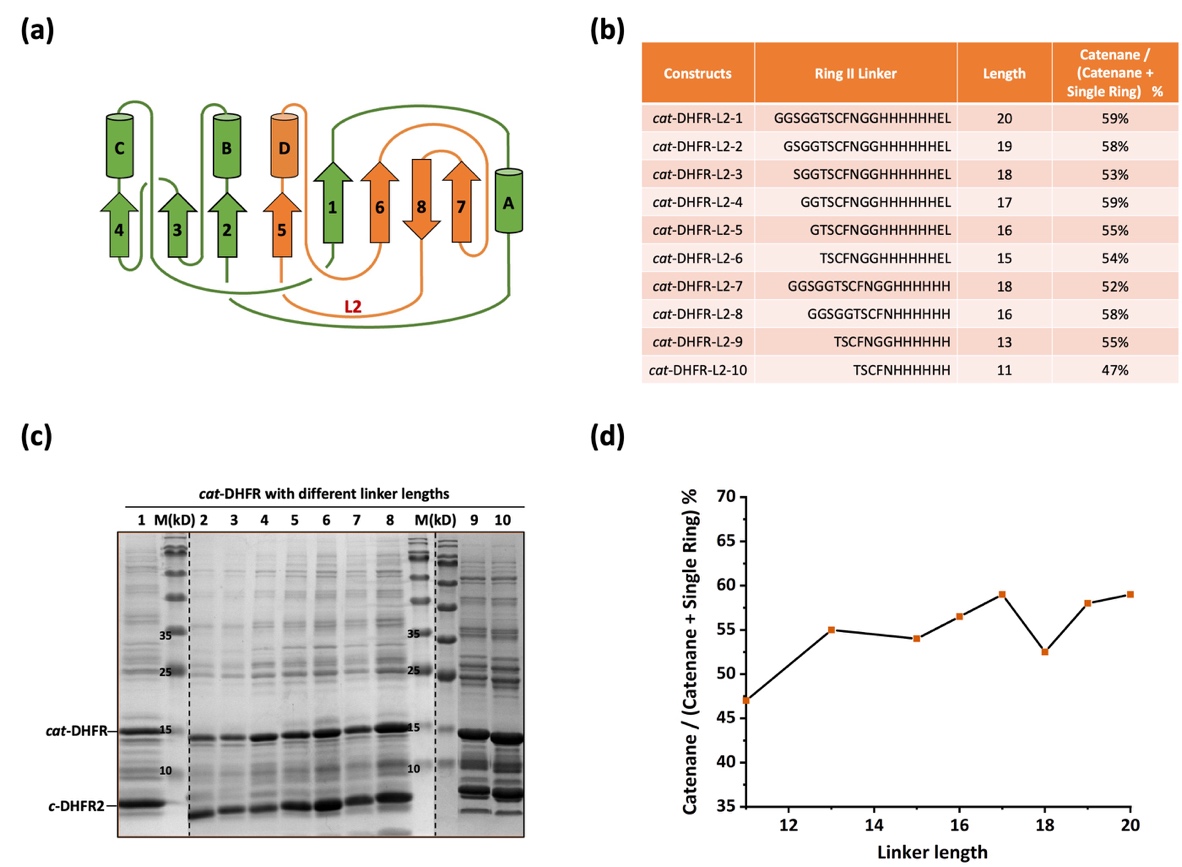


**Figure S4.** *cat-*DHFR with different lengths of the L2 flexible linker. (a) Schematic diagram of L2 in the *cat*-DHFR. (b) Specific amino acid sequences of the L2 and the percentage of concatenated products in the elution of different constructs. (c) SDS-PAGE analysis of the elution of *cat*-DHFR constructions. (d) The plot of the relationship between linker length and the percentage of catenane / (catenane + single ring).

**
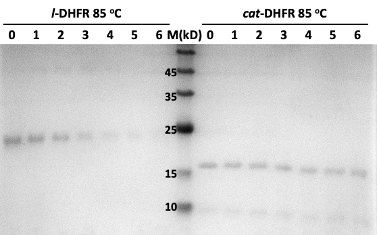
**

**Figure S5.** SDS-PAGE analysis of *l*-DHFR and *cat-*DHFR under the incubation at 85 ^o^C for 1-6 h.

**
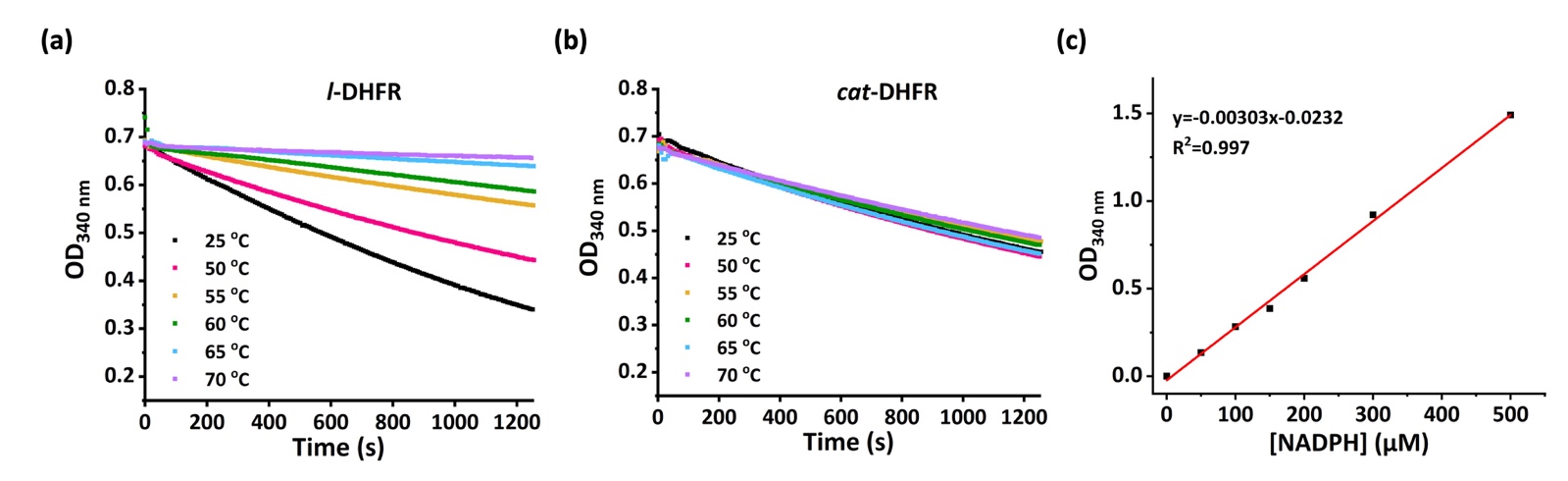
**

**Figure S6.** Kinetic curves of *l*-DHFR (a) and *cat-*DHFR (b) at 25 ^o^C and after incubation at 50 ^o^C, 55 ^o^C, 60 ^o^C, 65 ^o^C, and 70 ^o^C for 10 min. (c) Absorbance of NADPH solution with gradient concentration at 340 nm and a fitted plot as the standard curve.


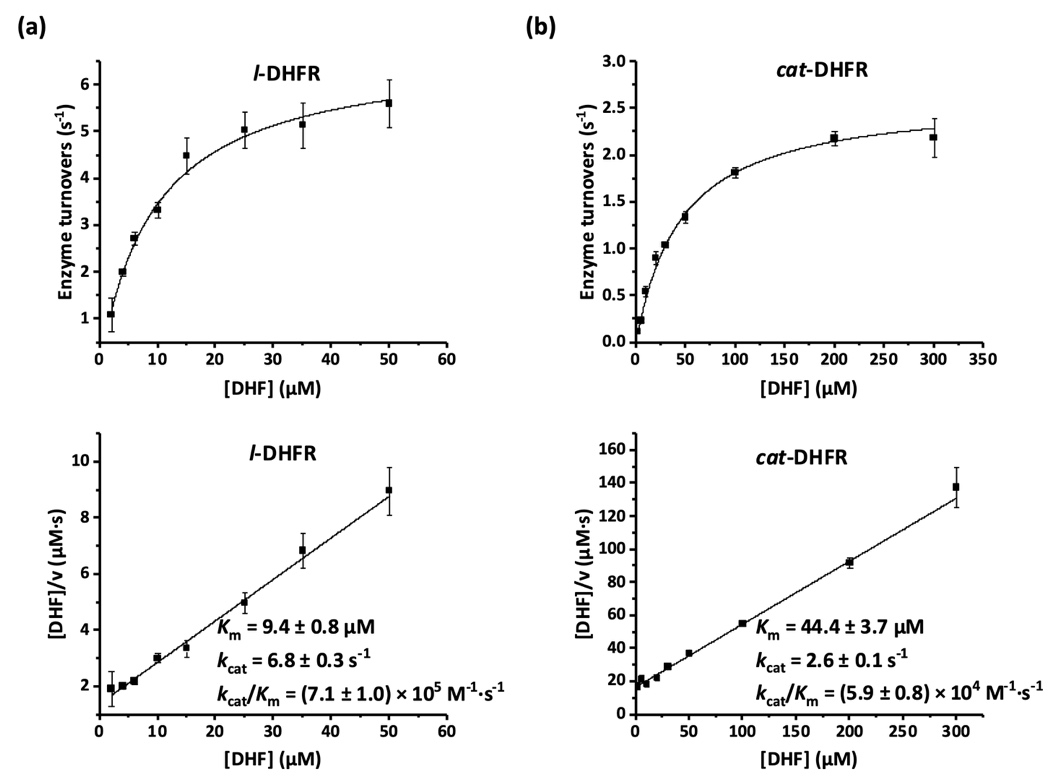


**Figure S7.** Michaelis curve and Hanes-Woolf plot of *l*-DHFR (a) and *cat*-DHFR (b).


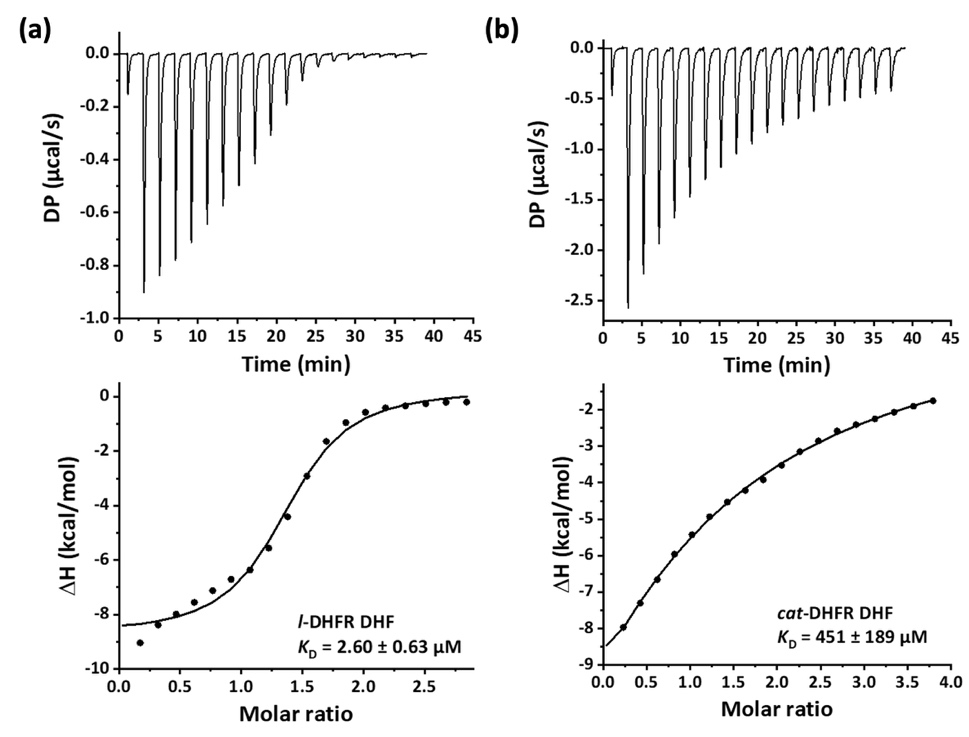


**Figure S8.** Isothermal titration calorimetry measurements for DHF binding to *l*-DHFR (a) and *cat*-DHFR (b).


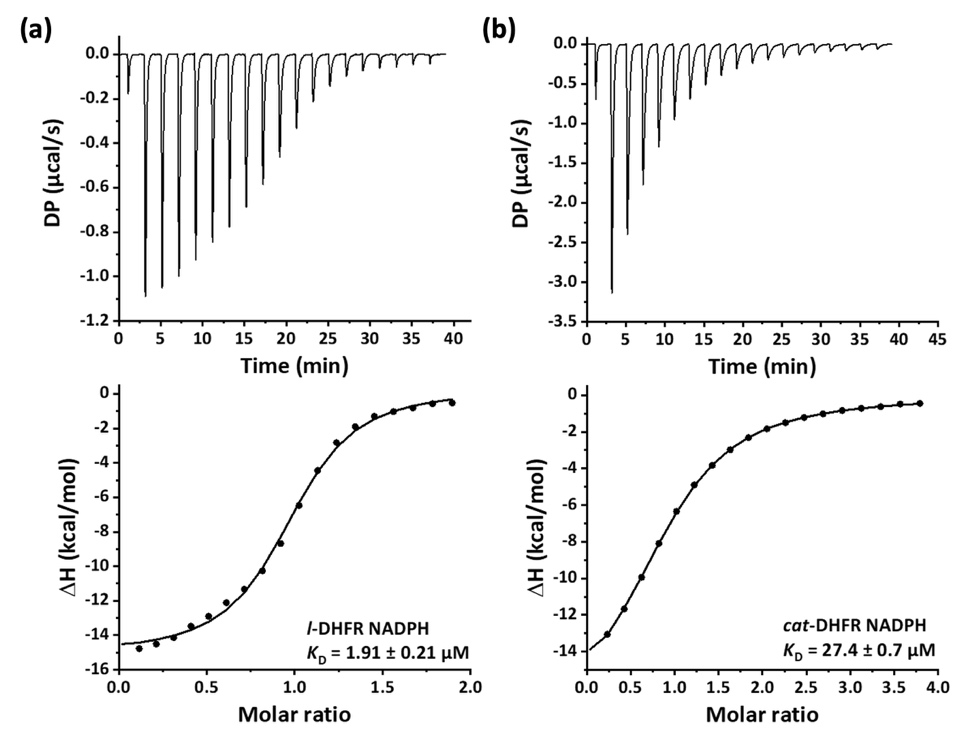


**Figure S9.** Isothermal titration calorimetry measurements for NADPH binding to *l*-DHFR (a) and *cat*-DHFR (b).


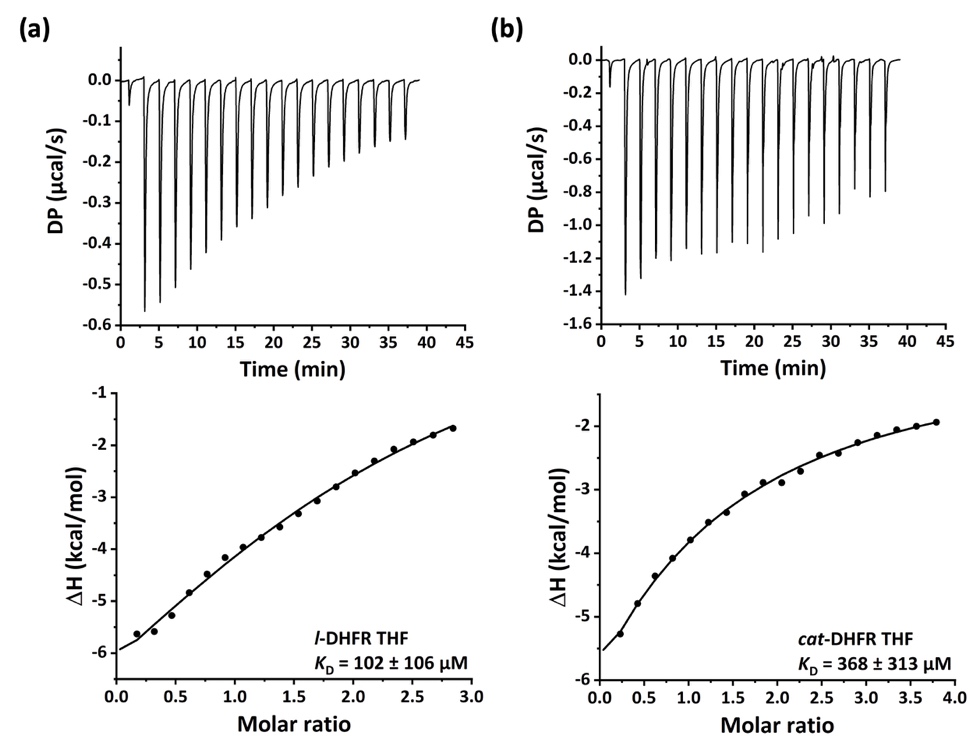


**Figure S10.** Isothermal titration calorimetry measurements for THF binding to *l*-DHFR (a) and *cat*-DHFR (b) in the presence of NADPH.


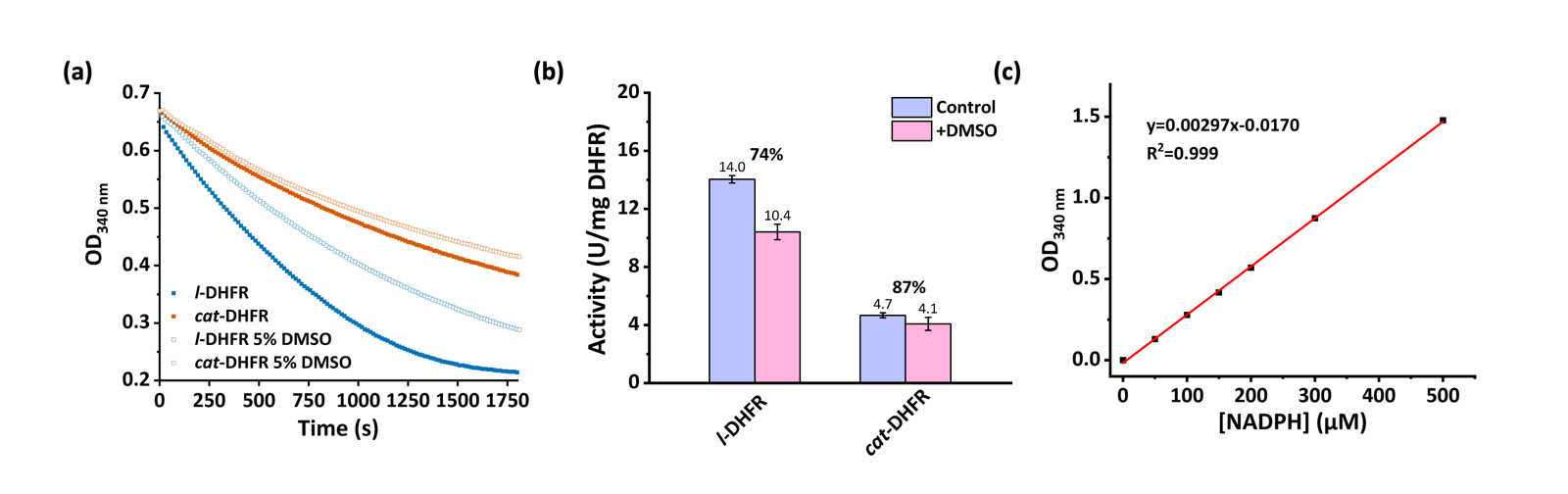


**Figure S11.** Kinetic curve (a), catalytic activities and percentage of activity preserved (b) of *l*-DHFR and *cat*-DHFR with/without the addition of 5% DMSO. 1 U·mg^-1^ DHFR is defined as the amount of enzyme that oxidizes 1.0 μmol of NADPH per minute, at pH 7.5 at room temperature per mg DHFR. (c) Absorbance of NADPH solution with gradient concentration at 340 nm and a fitted plot as the standard curve.

**References**

1. Baiesi M, Orlandini E, Trovato A *et al.* Linking in domain-swapped protein dimers. *Sci Rep* 2016; **6**: 33872.

2. Wang XW, Zhang WB. Protein catenation enhances both the stability and activity of folded structural domains. *Angew Chem Int Ed* 2017; **56**: 13985-9.

3. Liu Y, Duan Z, Fang J *et al.* Cellular synthesis and X-ray crystal structure of a designed protein heterocatenane. *Angew Chem Int Ed* 2020; **59**: 16122-7.

4. Liu Y, Bai X, Lyu C *et al.* Mechano-bioconjugation strategy empowering fusion protein therapeutics with aggregation resistance, prolonged circulation, and enhanced antitumor efficacy. *J Am Chem Soc* 2022; **144**: 18387-96.

5. Agah S, Poulos S, Yu A *et al.* Protein rethreading: A novel approach to protein design. *Sci Rep* 2016; **6**: 26847.

6. Eastman P, Swails J, Chodera JD *et al.* OpenMM 7: Rapid development of high performance algorithms for molecular dynamics. *PLoS Comput Biol* 2017; **13**: e1005659.

7. MacKerell AD, Bashford D, Bellott M *et al.* All-atom empirical potential for molecular modeling and dynamics studies of proteins. *J Phys Chem B* 1998; **102**: 3586-616.

8. Mathews CK, Scrimgeour KG, Huennekens FM. [48] Dihydrofolic reductase: Dihydrofolate + TPNH + H^+^ ⇄ tetrahydrofolate + TPN^+^. *Methods in enzymology*: Academic Press; 1963. 364-8.

9. Schoene C, Fierer JO, Bennett SP *et al.* SpyTag/SpyCatcher cyclization confers resilience to boiling on a mesophilic enzyme. *Angew Chem Int Ed* 2014; **53**: 6101-4.

10. Czekster CM, Vandemeulebroucke A, Blanchard JS. Kinetic and chemical mechanism of the dihydrofolate reductase from Mycobacterium tuberculosis. *Biochemistry* 2011; **50**: 367-75.

11. Stone SR, Morrison JF. Kinetic mechanism of the reaction catalyzed by dihydrofolate reductase from *Escherichia coli*. *Biochemistry* 1982; **21**: 3757-65.
